# State of well-being among residents in a tertiary center in Riyadh, Saudi Arabia

**DOI:** 10.1186/s12909-023-04596-4

**Published:** 2023-09-08

**Authors:** Fatimah Saeed AlAhmari, Alaa Aloqail, Shahad Almansour, Mohammad Bagha

**Affiliations:** 1 College of Medicine, Alfaisal university, Riyadh, Saudi Arabia; 2 Pediatrics Department, King Abdullah Specialized Children Hospital, Riyadh, Saudi Arabia

**Keywords:** Residency, Residents wellbeing, Subjective well-being, Job satisfaction

## Abstract

**Background:**

Medical residency is a part of postgraduate medical education and involves clinical training in a selected specialty. It is a challenging step in a physician’s professional development. This study aims to estimate the impact of the residency training program and demographic factors on the trainee’s state of well-being (SOW).

**Methods:**

This was an observational cross-sectional study carried out in the year 2019–2020, which aimed to measure the SOW of residents undergoing clinical training in Riyadh, Saudi Arabia. A total of 260 residents participated in the study. A self-administered validated online the World Health Organization, Quality of Life Scale questionnaire- BREF was distributed to collect the data. The collected information on four different domains was analysed and compared across the baseline characteristics and different specialties. When appropriate, the independent sample t-test, bivariate correlation analysis, and ANOVA tests were used.

**Results:**

A total of 260 resident responses were included in the final analysis The results revealed a significant difference in physical health scores (p = 0.006), social relationship scores (p = 0.038), and environmental scores (p < 0.001) while no significant difference was found in psychological health scores among the physicians’ specialties (p = 0.053). Post hoc comparison found statistically significant variations in the physical health domain between the medical and emergency specialties (p = 0.007), as well as surgical and emergency specialties (p = 0.024). There was also a significant difference between medical and emergency specialties (p = 0.008) in the social relationship domain. In the environment domain, significant variation was reported between medical specialties and emergency specialties (p = 0.001), as well asbetween surgical specialties and emergency specialties (p = 0.045). Female residents reported significantly lower quality of life in the physical (p = 0.020) and psychological (p = 0.032) domains.

**Conclusions:**

A significant relationship was found between physical, social, and environmental domains according to residents’ specialties. The factors that affected one or more domains included age, female gender, marital status, disease status, the number of on-calls received, and workload. We emphasize the importance of implementing policies to regulate working hours and on-call schedules as well as prioritizing mental health support.

## Introduction

Subjective well-being (SWB) refers to the people’s state to realize and evaluate their goals, presence of positive emotion, happiness, self-satisfaction that all originate from optimal functioning [1, 2]. It has an impact on human health in the long run, evidence showed that the lack or impairment of SWB can lead to different morbidities, including mental disorders [1]. Residents, health care workers under training, are exposed to emotional stress in their daily practice, including working under pressure, long working hours, sleep deprivation, and facing deaths of their patients. Therefore, they are more vulnerable to physical and mental disorders, which might compromise their SOW.

A previous systematic review conducted to identify causes associated with residents’ well-being, proved that autonomy, building of competence, strong social relatedness, enough sleep, and personal time are linked to better SWB [3]. Different studies in various countries evaluated the effect of residency training on trainees’ well-being. In a Nigerian study, 31% of the participants experienced emotional or mental health problems throughout their residency program [4]. Furthermore, 92% of trainees enrolled in 10 residency programs in England stated that work-related stress is moderate to severe and is negatively affecting their overall well-being [5]. In addition, in a study evaluating the predictors of well-being in residents at the University of Calgary in Canada, findings showed that regarding the overall well-being, no significant difference was seen between male and female groups, however, burnout was more presented in the female group [6]. Few other studies supported this theory [7, 8]. For instance, in a study conducted in Alberta, the females rated 40% more significant stress when compared to males 27% [8]. Unfortunately, no previous local study was conducted to address different domains of SWB in trainees of residency programs such as social relations, physical and mental health, achievement and spirituality. Nevertheless, multiple Saudi articles focused mainly on the burnout and stress during residency training.

Stress is a well-known factor for deteriorating the state of well-being on both physical and psychological levels [9]. Trainees from Saudi Arabia experience higher levels of stress when compared to their peers worldwide, reaching (84%) [10]. They also state their dissatisfaction with co-workers and training program [10]. Another Saudi study reported that 22.6% of residents from different specialties (Internal Medicine, Emergency Medicine, Family Medicine) had severe levels of stress [11]. Only one study assessed the social life satisfaction for pediatric residents in the eastern region in an attempt to test the hospital-learning environment that was reported to be “uninspiring” [12].

In this study we aim to estimate the impact of the residency program and demographic factors on the residents’ SOW. These findings can help to understand the current state of residents’ well-being and discover the characteristics of clinical training that could be improved to increase trainees’ satisfaction.

## Methods

### Study design and participants

This was a cross-sectional study conducted in a tertiary center, the King Abdul-Aziz medical city (KAMC), Ministry of National Guard-Health Affairs (MNG-HA), in Riyadh, Saudi Arabia. NGHA extended its mission to involve an academic dimension in 2004 by establishing the College of Medicine and the College of Nursing and Allied Medical Sciences. KAMC has passed the requirements for accreditation under the Joint Commission International (JCI) standards with excellent performance in December 2006. We included residents working in several departments of KAMC including pediatrics, internal medicine, surgery, family medicine, OB/GYN, dermatology, psychiatry, urology, orthopedics, ENT, ophthalmology, emergency medicine, anesthesia, neurosurgery, radiology, pediatric surgery, neurology and pediatric neurology [13, 14], who were willing to participate in this study. We excluded physicians/surgeons who were not practicing clinical work and residents who were rotating in other hospitals during the study.

### Sample size and sampling technique

RaoSoft, an online sample size calculator, was used to calculate the sample size from an approximate population of 811 residents, according to a 5% margin of error, 80% power of the study, and 95% confidence level. The sample size was calculated to be (260) residents (medical school graduates who are taking part in a graduate medical education (GME) program). The nature of the project design makes for a convenient sampling technique. This non-probability sampling approach voluntarily involved residents who were registered in residency programs at KAMC.

### Data collection and variables

In this study, we adopted a self-administered questionnaire: The World Health Organization Quality of Life Scale- BREF (WHOQOL Group, 1997). This tool is fully validated and designed to assess the quality of life based on the four main domains: physical health (7 items), psychological health (6 items), social relations (3 items), and environmental health (8 items). All these items were rated on a 5-point scale, with higher scores indicating better quality of life. Questions 3, 4, and 26 were inverted. The quality-of-life domain scores were calculated as usual by multiplying the mean domain score by a factor of 4, resulting in a range from 4 to 20 for each domain All domains were ethically approved to assess the respondent views in the context of their culture and value systems, and their personal goals, standards and concerns without any signs of discrimination. The four domains of the WHOQOL-BREF questionnaire were considered dependent variables and other data such as socio-demographics (sex, marital status, and history of medical illness) in addition to workload data (level of residence, number of on-call per month, extra-curricular activity, number of working hours and frequency of post-call time) were considered independent variables. The investigators distributed an anonymous self-administered online survey through email to all residents under KAMC residency program. The questionnaire was originally designed in English and no translation was needed since all residents are knowledgeable in English [14, 15].

### Ethical considerations

The study was approved by the IRB of the Ministry of National Guard-Health Affairs (Ref. # RC19/234/R).

### Statistical analysis

In this study, the data was analyzed using SPSS® version 26.0 (IBM Corporation, Armonk, NY, USA). The data is presented as the means ± standard deviations for continuous variables and as the proportions for categorical variables. Descriptive statistics analyses such as frequencies, percentages, means, and standard deviations, were used to measure demographic variables and workload characteristicsThe normality of the four domains was assessed using the Kolmogorov–Smirnov test. A Pearson’s correlation coefficient analysis was applied to determine the level of agreement between the four domains of the WHOQOL-BREF. An independent sample t-test was applied to assess the relationship between the four domains and workload and participant characteristics. Also, a one-way ANOVA with a post hoc test (Tukey) was conducted to compare the effect of physician specialization on the four domains of quality of life. Statistical significance was set at p < 0.05 for all variables assessed.

## Results

A total of 260 residents responded to a survey and completed the WHOQOL-BREF questionnaire in this study, with a response rate of 35%. In Saudi Arabia medical residency program is designed mainly for medical graduates who completed internship or have several years of general practice experience, thus most of the applicant are under 35. In our study the majority of residents aged between 20 and 30 (94.6%) years, the percentage of males was equal to females- 130 (50%). A total of 197 respondents (75.3%) were single, 36 (13.8%) reported a history of medical illness, 80.6% had the disease onset before enrolling on the residency program (Table 1).

**Table 1:** Participants’ socio-demographic characteristics

	N (%)
**Age**	
20–30	246(94.6)
30–40	14(5.4)
**Gender**	
Male	130(50)
Female	130(50)
**Marital Status**	
Single	197(75.8)
Married	63(24.2)
**History of medical illness**	
Yes	36(13.8)
No	224(86.2)
**Disease onset**	
Before residency	29(80.6)
During residency	7(19.4)

N: frequency, %: percentage

Table 2 shows the workload characteristics of residents; 158(60.8%) were in the first and second year of residency, while 102 (39.2%) were in the third and fourth year. The majority (91.5%) had a schedule with more than eight working hours per day for five days per week. Most of the post-call time was available after the morning shift (7am-12pm) (61.5%), followed by the afternoon shift(38.5%). Less than half of the respondents(41.9%) have engaged in an extra-curricular activity while they are still in the residency program.

**Table 2:** Residents’ workload characteristics

	N (%)
**Residency level**	
R1-R2	158(60.8)
R3-R4	102(39.2)
**Working hours**	
˂ 8	22(8.5)
≥ 8	238(91.5)
**Number of on-call per month**	
≤ 4	136(52.3)
> 4	124(47.7)
**Post-call time**	
Morning shift (Before 12 pm)	160(61.5)
Afternoon shift (After 12 pm)	100(38.5)
**Participate in any extra-curricular activity**	
Yes	109(41.9)
No	151(58.1)

N: frequency, %: percentage

**Fig. 1:**
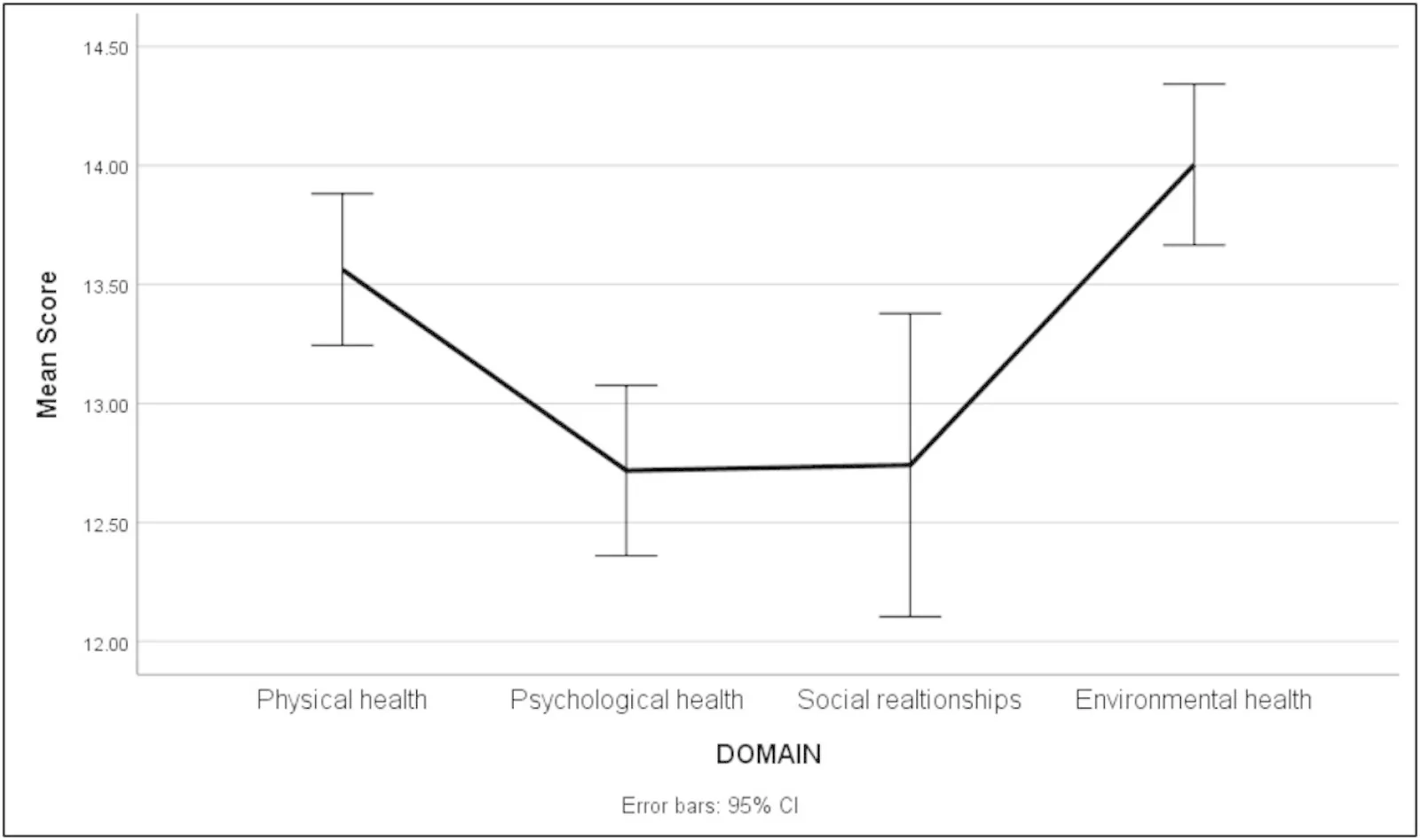
Distribution of the participants according to the mean scores of the four domains of the WHOQOL-BREF tool

Residents’ mean score regarding psychological health (domain 2) was the lowest among all domains with mean value of 12.72 ± 2.93, whereas physical health (domain1), social relationships (domain3), and environmental (domain4) domains reported mean scores of 13.56 ± 2.61, 12.88 ± 3.58, and 14 ± 2.77, respectively (Fig. 1). The mean overall quality of life score was 3.66 ± 0.88 and overall satisfaction with health was 3.32 ± 1.13 (Table 3).

**Table 3:** The scores of WHOQOL-BREF domains by resident

	Mean ± SD	Minimum	Maximum
**Ratings of participant’s quality of life**	3.66 ± 0.88	1	5
**Satisfaction with their health**	3.32 ± 1.13	1	5
**Physical health (Domain 1)**	13.56 ± 2.61	4	18.86
**Psychological health (Domain 2)**	12.72 ± 2.93	4	19.33
**Social relationships (Domain 3)**	12.88 ± 3.58	4	20
**Environmental health (Domain 4)**	14 ± 2.77	4	19.5

SD: standard deviation

**Fig. 2:**
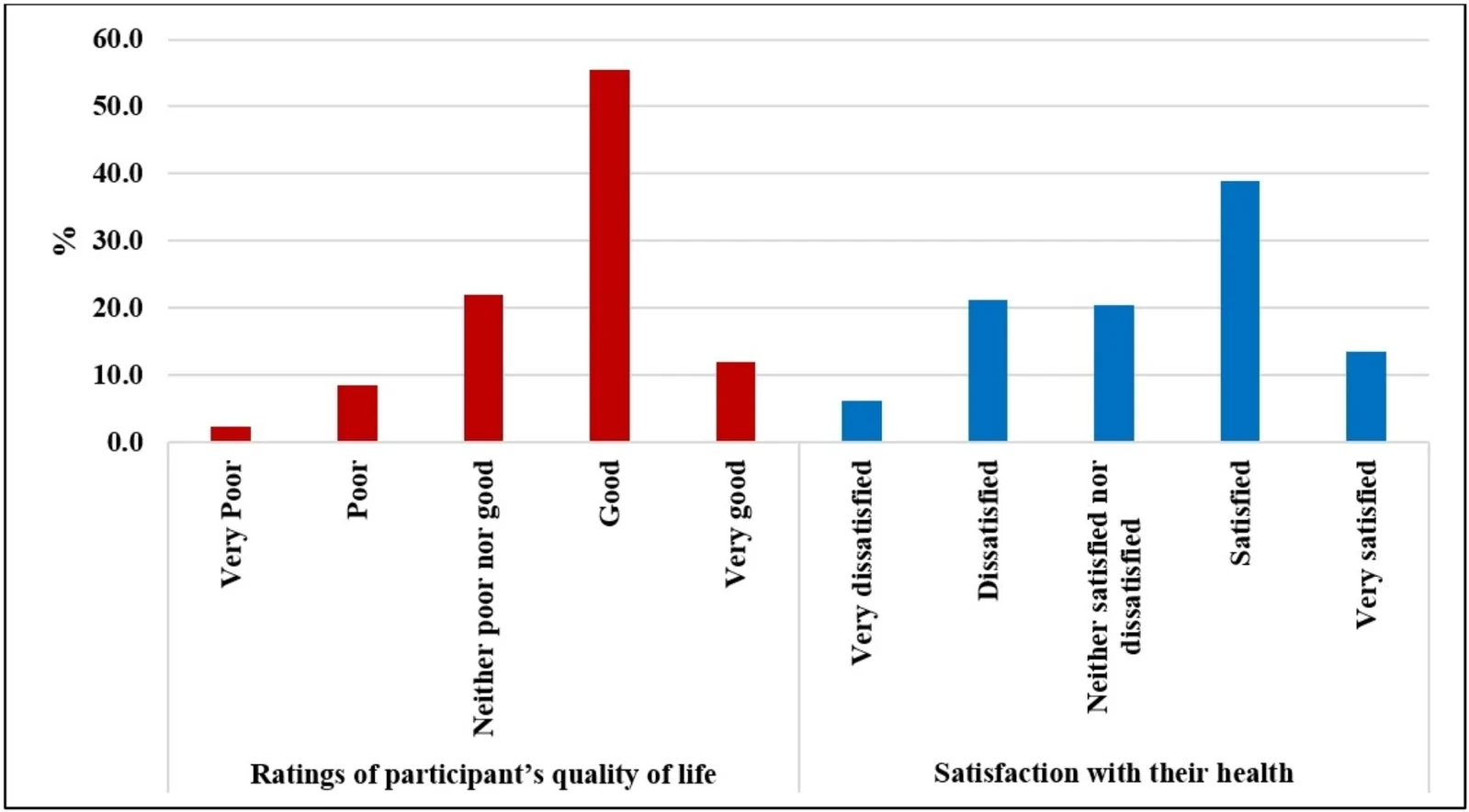
Percentage distribution of the participants according to their responses to ratings of quality of life and satisfaction with their health

More than half (67.3%) of residents reported good (good/very good) rate for their quality of life while 21.9% were neither poor nor good and only 10.8% reported poor (very poor/poor) rate. Residents’ responses towards the overall quality of life and overall satisfaction with life showed that there was a positive correlation with all the physical health (p < 0.001), psychological health (p < 0.001), social relationships (p < 0.001), and environmental domain scores (p < 0.001). It indicates that the resident’s overall quality of life and satisfaction with health increase when the scores of each of the four domains rise (Fig. 2) (Table 4).

**Table 4:** Correlation analysis between the two overall quality of life items (Q1 and Q2) and the four domains (domain1 – domain 4)

		Domain 1	Domain 2	Domain 3	Domain 4
**Q1**	*r*	0.55	0.609	0.476	0.542
	*P-value*	< 0.001	< 0.001	< 0.001	< 0.001
**Q2**	*r*	0.462	0.586	0.346	0.472
	*P-value*	< 0.001	< 0.001	< 0.001	< 0.001

r: correlation coefficient

Residents’ specialties were subdivided into three groups: general medical specialties, emergency specialties and surgical specialties. A one-way ANOVA was conducted to compare the effect of physician specialty on the four domains of quality of life. There was a significant difference in domain one score [F(2,257) = 5.190, p = 0.006] between different specialties, as well as in domain two and four, while no significant difference was found in third domain scores among thespecialties.Post hoc comparison using the Tukey test was carried out. There was a significant difference between medical and emergency specialties (p = 0.007) and between surgical specialties and emergency specialties (p = 0.024) in the physical health domain. There was also a significant difference between the medical and emergency specialties (p = 0.008) in the social relationship domain. In the fourth domain, environmental health, a significant difference was reported between the medical specialties and emergency specialties (p = 0.001) as well as surgical specialties and emergency specialties (p = 0.045) (Table 5).

**Table 5:** The scores of WHOQOL-BREF domains by physician specialty

			Mean ± SD	F (df)	p-value
Physical Health	Medicine	189	13.38 ± 2.66	5.190 (2,257)	**0.006**
	Surgery	30	13.10 ± 2.01		
	Emergency	41	14.73 ± 2.49		
Psychological Health	Medicine	189	12.50 ± 3.02	2.971 (2,257)	0.053
	Surgery	30	12.71 ± 2.67		
	Emergency	41	13.72 ± 2.52		
Social relationships	Medicine	189	12.54 ± 3.51	4.537 (2,257)	**0.012**
	Surgery	30	12.93 ± 3.84		
	Emergency	41	14.37 ± 3.41		
Environmental Health	Medicine	189	13.70 ± 2.83	7.284 (2.257)	**0.001**
	Surgery	30	13.92 ± 2.59		
	Emergency	41	15.48 ± 2.15		

SD: Standard deviation, df: degrees of freedom, F: F statistics

Post hoc test (Tukey tests):

**Physical health**: medical and emergency specialty (p = 0.007) and surgical specialty and emergency specialty (p = 0.024)

**Social relationship**: medical and emergency specialties (p = 0.008)

**Environmental health**: a significant difference was reported between the medical specialty and emergency specialty (p = 0.001) and a significant difference between the surgical specialty and emergency specialty (p = 0.045)

Males had significantly higher psychological (13.11 ± 2.77, p = 0.032) and physical health scores (13.94 ± 2.57, p = 0.020) than females (12.32 ± 3.05 and 13.19 ± 2.60, respectively). Married residents (14.39 ± 3.66, P = 0.001) had significantly higher score of their social relationship domain than single ones (12.39 ± 3.42). Residents with past medical history showed significantly lower mean scores in domain 1,2, and 4 when compared with others (Table 6).

**Table 6:** Comparison of the scores in the four domains according to socio-demographic variables

	Domain 1 (Mean ± SD)	Domain 2 (Mean ± SD)	Domain 3 (Mean ± SD)	Domain 4 (Mean ± SD)
**Gender**				
Male	13.94 ± 2.57	13.11 ± 2.77	12.81 ± 3.74	14.18 ± 2.78
Female	13.19 ± 2.60	12.32 ± 3.05	12.94 ± 3.42	13.82 ± 2.76
p-value	*t = 2.34, p =* ***0.020***	*t = 2.15, p =* ***0.032***	*t=-0.30, p = 0.764*	*t = 1.05, p = 0*.294
**Age**				
20–30	13.63 ± 2.61	12.78 ± 2.94	13.06 ± 3.50	14.09 ± 2.76
30–40	12.36 ± 2.33	11.33 ± 2.48	9.61 ± 3.51	12.35 ± 2.47
p-value	*t = 1.76, p = 0.078*	*t = 1.82, p = 0.069*	*t = 3.56, p =* ***0.003***	*t = 2.54, p =* ***0.023***
**Marital Status**				
Single	13.48 ± 2.58	12.58 ± 2.95	12.39 ± 3.42	13.94 ± 2.82
Married	13.82 ± 2.71	13.15 ± 2.86	14.39 ± 3.66	14.21 ± 2.61
p-value	*t=-0.91, p = 0.364*	*t=-1.36, p = 0.176*	*t=-3.9, p* ***< 0.001***	*t=-0.67, p = 0.506*
**History of medical illness**				
Yes	12.68 ± 2.65	11.74 ± 3.25	12.44 ± 3.65	13.01 ± 2.75
No	13.70 ± 2.58	12.88 ± 2.86	12.95 ± 3.57	14.16 ± 2.75
p-value	*t=-2.20, p =* ***0.029***	*t=-2.17, p =* ***0.031***	*t=-0.78, p = 0.436*	*t=-2.33, p =* ***0.021***

Table 7 shows a significant correlation between the number of on-calls and physical health (p < 0.001), psychological health (p = 0.003), social relationships (p = 0.008), and environmental domains (p < 0.001).Residents who received fewer than 4 on-calls had higher scores in these domains.Residents who worked more than 8 h had lower mean scores (12.63 ± 3.01, p = 0.016) in the psychological domain compared to the residents who worked less than 8 h (13.67 ± 1.69).

**Table 7:** Comparison of the scores in the four domains according to workload characteristics

	Domain 1 (Mean ± SD)	Domain 2 (Mean ± SD)	Domain 3 (Mean ± SD)	Domain 4 (Mean ± SD)
**Residency level**				
R1-R2	13.75 ± 2.66	13.00 ± 3.01	13.10 ± 3.44	13.99 ± 2.77
R3-R4	13.27 ± 2.51	12.29 ± 2.76	12.54 ± 3.78	14.02 ± 2.78
p-value	*t = 1.48, p = 0.141*	*t = 1.91, p = 0.057*	*t = 1.24, p = 0.218*	*t=-0.10, p = 0.923*
**Number of on-call per month**				
≤ 4	14.17 ± 2.29	13.23 ± 2.46	13.44 ± 3.19	14.67 ± 2.27
> 4	12.90 ± 2.78	12.16 ± 3.30	12.26 ± 3.88	13.27 ± 3.08
p-value	*t = 4.03, p* ***< 0.001***	*t = 2.96, p =* ***0.003***	*t = 2.6, p =* ***0.008***	*t = 4.10, p <* ***0.001***
**extra-curricular activity**				
yes	13.81 ± 2.66	12.95 ± 2.85	13.04 ± 3.47	14.29 ± 2.83
no	13.39 ± 2.57	12.55 ± 2.99	12.76 ± 3.66	13.80 ± 2.71
p-value	*t = 1.29, p = 0.197*	*t = 1.10, p = 0.271*	*t = 0.62, p = 0.534*	*t = 1.41, p = 0.159*
**Post-call time**				
Morning shift (Before 12 pm)	13.80 ± 2.65	12.93 ± 3.07	13.10 ± 3.75	14.23 ± 2.81
Afternoon shift (After 12 pm)	13.18 ± 2.51	12.37 ± 2.68	12.52 ± 3.27	13.64 ± 2.67
p-value	*t = 1.86, p = 0.064*	*t = 1.50, p = 0.135*	*t = 1.27, p = 0.204*	*t = 0.168, p = 0.094*
**Working hours**				
˂ 8	14.31 ± 1.84	13.67 ± 1.69	12.55 ± 2.39	14.48 ± 2.16
≥ 8	13.49 ± 2.66	12.63 ± 3.01	12.91 ± 3.67	13.96 ± 2.82
p-value	*t = 1.41, p = 0.160*	*t = 2.53, p =* ***0.016***	*t=-0.64, p = 0.524*	*t = 0.84, p = 0.403*

SD: standard deviation

## Discussion

We present the first study conducted in Saudi Arabia assessing the SWB of residents in multiple specialties. There was an equivalent percentagebetween both genders,which minimizes any gender-driven effects on the study results. The majority of residents in the population (60%) are in their early years of training (R1, R2), which is a typical period for them to adjust to their respective disciplines. It is important to consider that stress could have influenced their responses to the questionnaire, potentially acting as a confounding factor. Approximately (91.5%) of residents worked more than 8 h per day. These alarmingly long work hours might have a huge effect on the resident’s well-being and patient’s safety. Besides, Tahir et al. highlighted the absence of a policy adopted by the residency programs on the maximum number of working hours per week [16]. However, internationally it is recommended that the amount of working hours per week should not exceed 80 h. Our findings showed that almost 80% of residents felt that the duration of on-call is too long, and about two-thirds reported excessive stress due to the length of working hours and on-call [16]. In the current study, 38.5% reported a post-call time after 12pm or beyond 4pm. Furthermore, residents with more than 4 on-calls per month showed a lower score in the four domains compared to their colleagues with 4 or less on-calls. A systematic review demonstrated the negative effects of continuous duty periods on cognitive and clinical performance [17]. Most of the studies were in favor of fewer duty hours to minimize the adverse effects. Some of the studies have reported the negative impact on the longer duration of shift on the physical health as the physicians are prone to motor vehicle accidents [18–20]. However, a review of residents’ studies by Reed et al. has shown that residents should define the ideal shift length on their own [17].

The overall state of well-being was evaluated in all the four domains of quality of life (Domain 1: Physical Health, Domain 2: Psychological Health, Domain 3: Social Relationships, Domain 4: Environment).Compared to the other domains, the mean score in psychological health (domain 2) was the lowest, followed by social relationships (domain 3). It could be due to the long working hours and commitment toward their patients and other residency obligations that limit their time in social engagements and gatherings. A previous systematic review had similar results, where residents reported improved quality of life in caseof getting adequate rest, more time for family and for socializing [21]. More than half of residents reported a (good/very good) rate for their quality of life and around 11% reported it to be poor. These disparities may reflect physicians’ high self-esteem, the amount of income, or the cultural influence and the vision of the physician in Saudi Arabia. The residents who reported poor quality of life might suffer fromdepression and lack of sleep.

Among physicians with different specialties, residents who work in surgical specialties scored the lowest in their physical health domains compared to other residents. The amount of time spent on-call, the physical and emotional stress are all factors contributing to their health. A large study of 582 surgeons trained at the University of Michigan-Ann Arbor found that 4% of the sample group suffered from a low sense of personal achievement whereas 32% showed elevated levels of emotional fatigue and 13% with elevated levels of depersonalization [22]. Interestingly, in our study residents in different specialties had no substantial differences in the psychological domain. However, residents in medical specialties have reported lower psychological health scores. Unfortunately, there is insufficient effort and attention towards physicians’ mental health despite the evidence of a high rate of untreated mood disorders. In a prospective study of more than 1300 male physicians from The Johns Hopkins University, the lifetime prevalence of clinically significant depression was 12.8% [23]. Whereas the lifetime rates of depression among physicians tend to be close to those in the general population, suicide is disproportionately high in rates as a cause of mortality in physicians [24]. Depression frequently stays unrecognized or untreated until the emotional suffering of a doctor undermines his or her ability to take care of patients. Physicians requesting assistance often receive a punitive response, including discrimination in medical licenses, hospital privileges, and professional development. Therefore, while doctors may be more open to medical services for depression treatment, they face challenging regulatory and occupational obstacles that may prevent them from seeking assistance. Residents of Emergency medicine scored the highest in regards to the environment quality of life domain. This may be a reflection of the flexibility of shifts and working hours. Similarly, a previous study found that residents of flexible duty-hour systems noted various advantages in regard to near all aspects of patient safety, quality of treatment, surgical training, and professionalism [25].

In addition, in a study evaluating the predictors of well-being in residents at the University of Calgary in Canada, it was proved that regarding overall well-being, no significant difference was seen between the male and female groups [6]. However, burnout was more prevalent in females. For instance, in a study conducted in Alberta, females rated 40% more significant stress when compared to males reaching 27% [8]. Our study results indicate that female residents had significantly lower quality of life in the physical and psychological domains. The lower quality of life experienced by female residents in the physical and psychological domains may be attributed to societal expectations, work-related challenges, academic stress, and a lack of support and mentorship. Previous studies on medical education have also revealed that female medical students experienced higher levels of academic stress and worse well-being. Addressing these issues requires creating inclusive environments, promoting work-life balance, and offering support programs tailored to the specific needs of female residents.

The suicide mortality ratio of male doctors is almost 1.5- to 3.8-fold higher than that of other professions [24]. Among female doctors, the ratio is even higher, with a 3.7- to 4.5-fold increased risk of a suicide death [26, 27]. Married residents showed a higher score compared to singles in terms of psychological health domain. Despite the stacked clinical and education responsibilities, most married residents have stated a dramatical increase in the sense of how predictable and manageable the schedule is. This sense of control, even with a too-busy schedule, must promote the settled feelings. In a study evaluating the effect of 80- hours shifts on marriage and childbirth, the parental status frequency increased from 27 to 43% as the perception of life-control within the work-hour restrictions rose [28]. Having a stable insight and controllable time are thought to be the factors of psychological health in married individuals.

Our research has some limitations. Response rate was below our expectations, despite the fact that we sent reminders, tried to prompt the trainees and remind them about the time of morning meetings. Furthermore, not all residency programs have the same oncall and post-call regulations, hence it was hard to meet the team leaders and ask them for their support. Additionally, as it isa cross sectional study, we needed all responses within certain time, and responding to our research was not top priority for residents which can compromise the responses received.

The findings from the study suggest that long working hours, excessive on-call duration, and specialty-specific challenges have a negative impact on the well-being of residents. The study emphasizes the importance of implementing policies to limit working hours and reduce on-call shifts to improve the quality of life and patient safety. It also highlights gender differences in burnout rates and the need for gender-specific support. Furthermore, we underscore the significance of mental health support for healthcare professionals and the benefits of flexible duty-hour systems. These findings provide valuable insights applicable to the general population, emphasizing the need to prioritize work-life balance, address specialty-specific challenges, and promote mental well-being in healthcare settings worldwide.

## Conclusion

All four domains were studied to address the status of well-being among Saudi residents. A significant relationship was found between physical, social, and environmental domains according to residents’ specialties. A low score was reported in the psychological domain among the medical specialties, whereas the emergency specialty was found to have higher physical health, social relationships, and environmental domain scores compared to other specialties. Female residents reported significantly lower quality of life in the physical and psychological domains. The other factors that affected one or more domains included age, marital status, disease status, number of on-call hours and workload. A large multi-center study of resident well-being and its effect on patient safety is needed to develop a better work environment.

### Recommendations

Based on our study, we recommend implementing policies to regulate working hours and reduce on-call shifts, prioritizing mental health support for residents, addressing specialty-specific challenges, promoting flexible duty-hour systems, and improving data collection methods. These measures aim to enhance the well-being of residents, particularly considering the negative impact of long working hours, gender differences in burnout rates, and the need for tailored support. By prioritizing work-life balance, mental health, and specialty-specific challenges, healthcare institutions can create a healthier and more supportive environment for residents, ultimately benefiting both their well-being and patient care.

## Data Availability

All the data is provided in the manuscript. Further, the survey can be provided through request to the corresponding author’s email address.
